# Apical Vertebras Distribution Modifier for Coronal Balance Classification in Adult Idiopathic Scoliosis

**DOI:** 10.3390/jpm13060897

**Published:** 2023-05-26

**Authors:** Aixing Pan, Yong Hai, Lawrence G. Lenke, Zhaomin Zheng, Jincai Yang

**Affiliations:** 1 Beijing Chaoyang Hospital, Capital Medical University, Beijing 100020, China; 2 New York-Presbyterian Och Spine Hospital, New York, NY 10034, USA; 3 The First Affiliated Hospital, Sun Yat-sen University, Guangzhou 510062, China

**Keywords:** scoliosis, coronal balance, classification, apical vertebras

## Abstract

**Background:** We aimed to propose the apical vertebras distribution modifier to supplement the coronal balance (CB) classification for adult idiopathic scoliosis (AdIS). An algorithm to predict postoperative coronal compensation and avoid postoperative coronal imbalance (CIB) was proposed. **Methods:** Patients were categorized into CB and CIB groups according to the preoperative coronal balance distance (CBD). The apical vertebras distribution modifier was defined as negative (−) if the centers of the apical vertebras (CoAVs) were on either side of the central sacral vertical line (CSVL) and positive (+) if the CoAVs were on the same side of the CSVL. **Results:** A total of 80 AdIS patients, with an average age of 25.97 ± 9.20 years, who underwent posterior spinal fusion (PSF) were prospectively recruited. The mean Cobb angle of the main curve was 107.25 ± 21.11 degrees at preoperation. The mean follow-up time was 3.76 ± 1.38 (2–8) years. At postoperation and follow-up, CIB occurred in 7 (70%) and 4 (40%) CB− patients, 23 (50%) and 13 (28.26%) CB+ patients, 6 (60%) and 6 (60%) CIB− patients, and 9 (64.29%) and 10 (71.43%) CIB+ patients. Health-related quality of life (HRQoL) was significantly better in the CIB− group compared with that of the CIB+ group in the dimension of back pain. To avoid postoperative CIB, the correction rate of the main curve (CRMC) should match the compensatory curve for CB−/+ patients; the CRMC should be greater than the compensatory curve for CIB− patients; and the CRMC should be less than the compensatory curve for CIB+ patients, and the inclination of the LIV needs to be reduced. **Conclusions:** CB+ patients have the least postoperative CIB rate and the best coronal compensatory ability. CIB+ patients are at a high risk of postoperative CIB and have the poorest coronal compensatory capacity in the event of postoperative CIB. The proposed surgical algorithm facilitates the handling of each type of coronal alignment.

## 1. Introduction

Idiopathic scoliosis (IS) is the most common etiological type of scoliosis. Adult idiopathic scoliosis (AdIS) is, in essence, a continuation of adolescent idiopathic scoliosis (AIS). Spine curvature of an idiopathic nature that began during teenage years may progress during adult life. AdIS has more symptoms than AIS because of degeneration in discs and joints. Adult patients may have a variety of symptoms, which can lead to gradual loss of function, such as low back pain, fatigue, and radiating pain caused by nerve compression.

Posterior selective fixation and spinal fusion remain the mainstay of surgical treatment for scoliosis at present [1]. The goals of surgery are to restore spinal balance and reduce pain and discomfort and maintain corrected alignment by fusing and stabilizing the spinal segments. Optimizing the correction of the coronal plane deformity and balancing the trunk are the primary goals of PSF surgery for young adults.

Coronal balance (CB) of the spine can be accurately assessed and measured on standing whole spine radiographs. The most commonly used imaging parameter to assess CB is the distance between the C7 plumb line (C7PL) and the central sacral vertical line (CSVL), defined as coronal balance distance (CBD). However, CBD does not fully represent the full picture of patients’ coronal balance, especially for patients with severe curvature. In some patients with severe scoliosis, although the CBD is normal, the center of gravity of the patient’s trunk is shifted, resulting in an imbalanced coronal plane. Even with an equally abnormal CBD, the coronal imbalance (CIB) between patients can be different due to the individualized curvature shape and position.

Therefore, the compensatory mechanism of the CB is far more complex than what we currently know about it, especially in patients with severe and rigid scoliosis [2,3]. Ploumis et al. [4] highlighted the problem of CIB in adult patients with spinal deformity after surgical correction. Some post-surgical CIB is temporary, but some is permanent. In addition, some of the post-surgical CB can be maintained over time, but some is gradually lost. According to Daubs et al. [5], an incidence of 11.8% of adult scoliosis patients demonstrated a loss of coronal balance of more than 10 mm following PSF surgery. Furthermore, CIB has been reported to be strongly associated with a decrease in health-related quality of life (HRQoL) outcomes [6].

**Figure 1:**
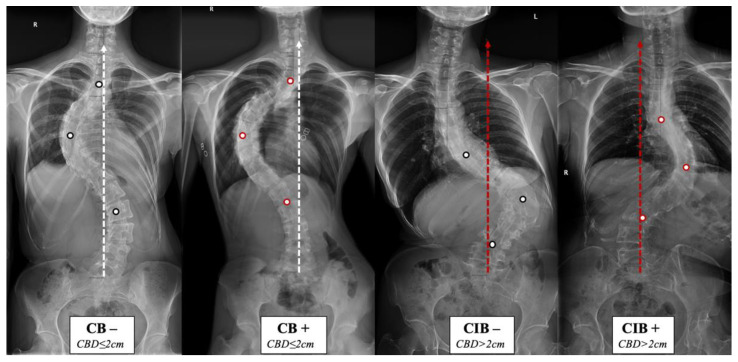
Coronal balance classification of adult idiopathic scoliosis according to the standing anterior-posterior whole spine radiograph. The apical vertebras distribution modifier (+/−) is introduced to supplement the previous classification based on the coronal balance distance (CBD). CB is diagnosed if CBD ≤ 2 cm, and CIB is diagnosed if CBD > 2 cm. The apical vertebra modifier is defined as negative (−) when CoAVs are on either side of the CSVL. The apical vertebra modifier is defined as positive (+) when CoAVs are on the same side of the CSVL. The white dots represent CoAVs, and the dotted arrows represent the CSVL.

**Figure 2:**
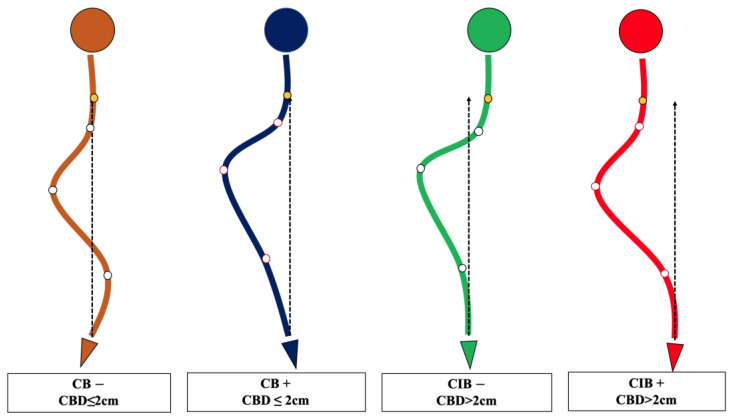
Schematic diagram of the novel coronal balance classification for adult idiopathic scoliosis. CB is diagnosed if CBD ≤ 2 cm, and CIB is diagnosed if CBD > 2 cm. The apical vertebra modifier is defined as negative (−) when CoAVs are on either side of the CSVL. The apical vertebra modifier is defined as positive (+) when CoAVs are on the same side of the CSVL. The yellow dot represents the center of C7, and the white dots represent CoAVs. The dotted arrows represent CSVL.

The lack of understanding of the natural history and compensatory mechanism of CB/CIB inspired us to propose the apical vertebras distribution modifier to supplement the CB classification for AdIS with a severe curve, which is defined as Cobb angle > 80 degrees in this study [7,8,9,10] (Figure 1 and Figure 2). A corresponding surgical algorithm is also proposed to address each type of coronal alignment. Surgical strategies were suggested in the algorithm according to the preoperative coronal classification to minimize the incidence of postoperative CIB.

## 2. Methods

### 2.1. Patient Selection

A single-center prospective cohort study was performed for adult idiopathic (AdIS) patients who underwent PSF surgery, which was approved by the Clinical Research Ethics Committee of our hospital. The main inclusion criteria were (a) patients diagnosed with adult idiopathic scoliosis with a main curve Cobb angle > 80 degrees; (b) no history of spinal surgery; and (c) postoperative follow-up of more than 24 months.

Clinical demographic data were collected and analyzed, including age at the initial operation, gender, height, weight, body mass index (BMI), and surgical information including the level of fusion. Health-related quality of life (HRQoL) related instruments were completed at the final follow-up, including the Oswestry Disability Index (ODI) and Visual Analogue Scale (VAS) (where 0 represents no pain, and 10 represents the most severe pain), the Short Form 36 (SF-36), and SRS-22 (Scoliosis Research Society) patient questionnaire.

Standing anterior-posterior full spine radiograph was obtained before surgery, after surgery, and at the last follow-up visit. Coronal balance distance (CBD) was defined as the distance between the C7 plumb line (C7PL) and the central sacral vertical line (CSVL). CBD ≤ 2 cm is the most commonly used diagnostic criterion in numerous published articles for CB [11,12,13,14]. Thus, CB was diagnosed if CBD ≤ 2 cm, and CIB was diagnosed if CBD > 2 cm.

The apical vertebras distribution modifier is proposed in this study to supplement the coronal balance classification based on the positional relationship between the centers of the apical vertebras (CoAVs) and the CSVL (Figure 1 and Figure 2). The apical vertebra modifier is defined as negative (−) when CoAVs are on either side of the CSVL. The apical vertebra modifier is defined as positive (+) when CoAVs are on the same side of the CSVL.

Therefore, we could categorize the patients into four groups, CB−/+ and CIB−/+. All patients were classified by the two attending surgeons according to the classification method described above. The interval between the two classifications was 1 week. In addition, for cases in which the two observers held different classification opinions, a third observer, a senior spine surgeon, was involved in joint judgment and to determine the final classification. The consistency of classification was judged based on the Kappa value of the consistency test.

### 2.2. Statistical Analysis

All statistics were performed using the software SPSS Statistics Version 23 (IBM, Armonk, New York, NY, USA). Paired or independent student’s *t*-test was used to analyze continuous data. The chi-square test was used to analyze enumeration data. AP-value less than 0.05 was considered statistically significant.

## 3. Results

A total of 80 (22 male and 58 female) AdIS patients were included. The average age at the operation was 25.97 ± 9.20 years. The mean follow-up time was 3.76 ± 1.38 (2–8) years. The mean Cobb angle of the main curve was 107.25 ± 21.11 degrees. The mean preoperative CBD was 20.05 ± 21.36 mm. The demographic and radiological measurements of the patients are summarized in Table 1, and the distribution of upper instrumented vertebrae (UIV) and lowest instrumented vertebrae (LIV) is shown in Supplemental Table S1.

The Kappa value of the consistency test between classifications made by different observers is 0.959, and the Kappa value of the consistency test between classifications made by the same observer is 0.979, indicating that the classification method has high inter-observer and intra-observer consistency (Supplemental Tables S2 and S3).

Among the included patients, there are 10 (12.5%) CB− patients, 46 (57.5%) CB+ patients, 10 (12.5%) CIB− patients, and 14 (17.5%) CIB+ patients according to the coronal classification rules. The comparison of demographic data and radiological parameters between the four groups is shown in Table 2. Patient age, gender, follow-up time, and Cobb angle of pre/postoperative principal curvature did not differ significantly between the groups (Table 1).

In the CB− group, CIB occurred in 7 (70%) and 4 (40%) patients at postoperation and follow-up, respectively. In the CB+ group, 23 (50%) and 13 (28.26%) patients had CIB at postoperation and follow-up, respectively. In the CIB− group, CIB occurred in 6 (60%) patients at postoperation and follow-up. In the CIB+ group, CIB occurred in 9 (64.29%) and 10 (71.43%) patients at postoperation and follow-up, respectively (Table 3). The results indicate that preoperative CB+ patients have the best compensatory ability during the postoperation follow-up. However, preoperative CIB+ patients have the poorest compensatory capacity in the event of postoperative CIB.

HRQoL was assessed at the last follow-up for the four groups of patients, including visual analogue score (VAS) for back/leg pain, Oswestry Disability Index (ODI), SRS-22 (Table 4), and Short Form-36 (Table 5). CIB− patients’ HRQoL was significantly better than that of CIB+ patients at the last follow-up in terms of the VAS back pain score (0.67 ± 0.94 vs. 2.00 ± 1.30, *p* = 0.01) and the pain dimensions assessed by the SRS-22 patient questionnaire (4.0 ± 0.36 vs. 4.5 ± 0.43, *p* = 0.01). However, patients in different groups did not differ significantly in lower extremity pain, physical function, social function, general health, mental health, etc.

**Figure 3:**
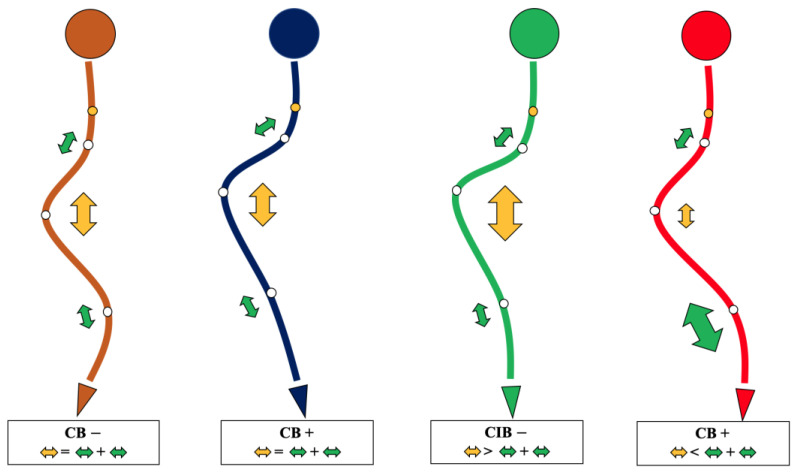
The surgical algorithm for each type of coronal balance. The yellow arrow indicates thoracic curvature correction, and the green arrows indicate upper thoracic and thoracolumbar curvature correction. The larger the arrow, the greater the correction.

**Figure 4:**
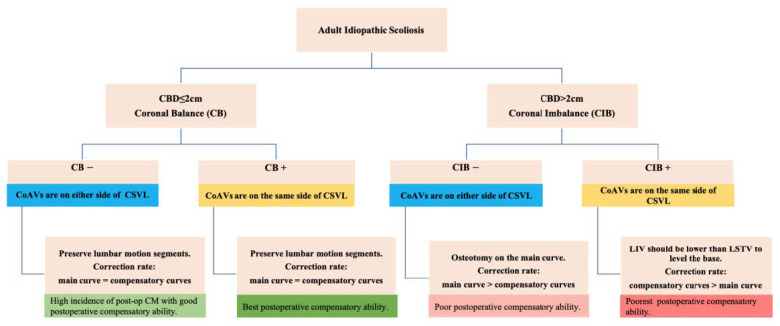
Schematic diagram of the correction strategy for each type of patient. Firstly, CB is diagnosed if CBD ≤ 2 cm, and CIB is diagnosed if CBD > 2 cm. Secondly, the apical vertebra modifier is defined as negative (−) when CoAVs are on either side of the CSVL and positive (+) when CoAVs are on the same side of the CSVL. Thirdly, the surgical correction strategies for the four types of patients, as well as the postoperative compensatory capacities, are illustrated in the bottom line of the figure. Preoperative CB+ patients have the best compensatory ability in the event of postoperative CIB. Preoperative CIB+ patients have the poorest postoperative compensatory capacity.

**Figure 5:**
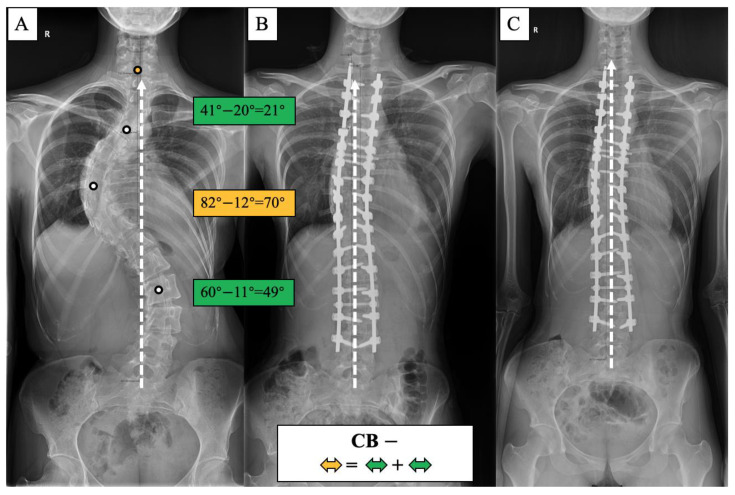
CB− case, 23-year-old female AdIS patient ((**A**): preoperation, (**B**): postoperation, and (**C**): 2-year follow-up). The main thoracic curve was corrected from 82° to 12° (70° correction). The upper thoracic curve was corrected from 41° to 20° (21° correction), and the thoracolumbar curve was corrected from 60° to 11° (49° correction). The correction rate of the main curve matched the compensatory curves, and the coronal plane balance was maintained after surgery and long-term follow-up.

**Figure 6:**
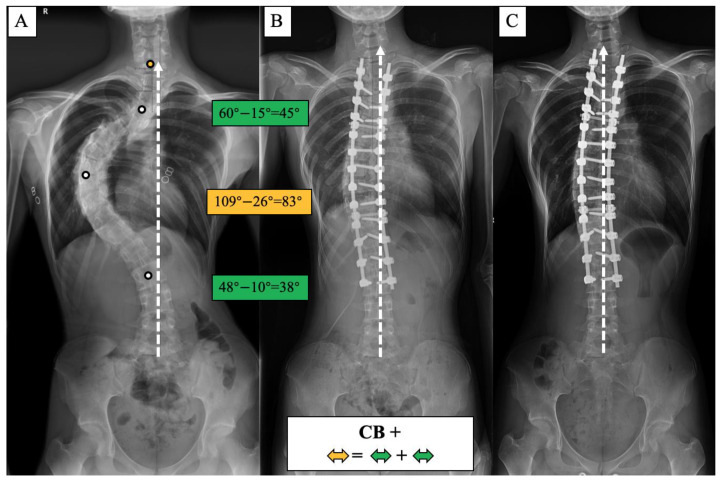
CB+ case, 25-year-old female AdIS patient ((**A**): preoperation, (**B**): postoperation, and (**C**): 3-year follow-up). The main thoracic curve was corrected from 109° to 26° (83° correction). The upper thoracic curve was corrected from 60° to 15° (45° correction), and the thoracolumbar curve was corrected from 48° to 10° (38° correction). The correction rate of the main curve matched the compensatory curves, and the coronal plane balance was maintained after surgery and long-term follow-up.

**Figure 7:**
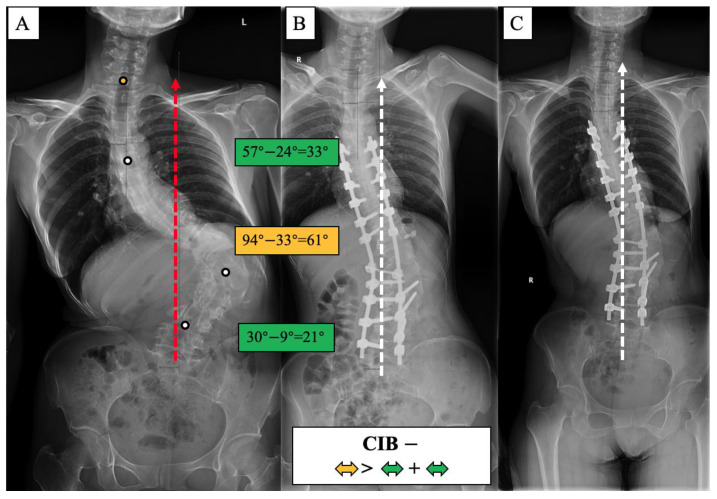
CIB− case, 27-year-old female AdIS patient ((**A**): preoperation, (**B**): postoperation, and (**C**): 2.5-year follow-up). The main thoracolumbar curve was corrected from 94° to 33° (61° correction). The thoracic curve was corrected from 57° to 24° (33° correction), and the lumbar-sacral curve was corrected from 30° to 9° (21° correction). The correction rate of the main curve was greater than the compensatory curves. The coronal plane balance was improved after surgery, and further improvement could be observed at the final follow-up.

**Figure 8:**
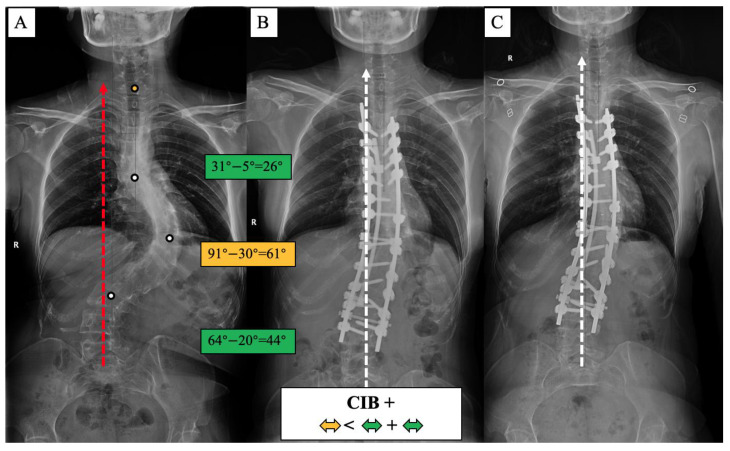
CIB+ case, 21-year-old female AdIS patient ((**A**): preoperation, (**B**): postoperation, and (**C**): 2-year follow-up). The main thoracic curve was corrected from 91° to 30° (61° correction). The upper thoracic curve was corrected from 31° to 5° (26° correction), and the lumbar curve was corrected from 64° to 20° (44° correction). The correction rate of the main curve was less than the compensatory curves. The coronal plane balance was improved after surgery, but no further improvement could be observed at the final follow-up.

A surgical algorithm was proposed based on the CB classification to avoid postoperation CIB (Figure 3). In addition, a schematic diagram of the correction strategy for each type of patient is shown in Figure 4. For CB+ patients, the correction rate of the main curve (CRMC) should match the compensation curve. For CB− patients, the CRMC should match the compensatory curve. Most of the lumbar motion segments can be retained to provide optimal compensatory ability. However, the postoperative compensatory capacity of CB− patients is not as good as CB+ patients due to the limited mobility of the preserved lumbar motion segments. For CIB− patients, the CRMC should be greater than the compensatory curves. Osteotomy at the concave side of the main thoracolumbar curve is recommended. Multiple-level asymmetrical Ponte osteotomy is a safe and effective technique to improve the flexibility of the spine as well as the correction rate of rigid adult idiopathic scoliosis [15]. For CIB+ patients, the CRMC should be less than the compensatory curves, and the LIV should be kept even during the surgery. Preserving lumbar mobility is not the primary consideration when making a surgical plan for CIB+ patients. Typical cases are shown in Figure 5, Figure 6, Figure 7 and Figure 8.

## 4. Discussion

The importance of sagittal alignment in scoliosis surgery to improve patients’ HRQoL and reduce complications of internal fixation has been well documented in the last decade [16,17]. However, relatively few studies have focused on coronal alignment. Previous studies have reported that postoperative CIB is associated with back pain, unsatisfactory appearance, poorer HRQoL, and even revision surgery [18,19,20]. The occurrence of postoperation CIB is multifactorial, with potentially relevant factors including improper selection of fusion segment, over-correction or under-correction, pelvis tilt, and lower limbs discrepancy [14,21], resulting in the compensation mechanism being complex and unpredictable.

Traditionally, coronal trunk balance is defined as CBD < 2 cm [14]. However, due to the complexity of scoliosis, the CBD alone cannot fully represent the coronal balance characteristics or provide complete and specific guidance for surgical strategies. Thus, we introduce the apical vertebra distribution modifier, which is the position relation between the CoAVs and CSVL. In addition, a novel coronal balance classification for AdIS was proposed to classify coronal balance more comprehensively and provide guidance for surgical treatment. The classification proposed in this study is proved to be well consistent and easy to use in the clinic.

Patients with congenital scoliosis, degenerative scoliosis, and syndromic scoliosis and those who had sacral-pelvic fixed were excluded from this study. The included patients did not differ significantly between groups in terms of age, gender, severity of the curve, distal unfused motor segments, and duration of follow-up. Thus, the included patients had similar demographic characteristics and surgical interventions between groups.

The incidence of postoperative CIB was 50% in CB+ patients and 70% in CB− patients, which was the highest among the four groups. The majority of postoperative CIB in this case series was caused by a mismatch in the correction rate between the primary and compensation curves. Nearly half of the postoperative CIB was compensated to CB at the final follow-up, benefiting from the adjustment of the LIV inclination angle. The decompensation or CIB rate (CBD > 2cm) in the final follow-up was 28.26% and 40% in CB+ and CB− patients, respectively, higher than the 16.83% reported by Miller for 908 patients with AIS with a smaller preoperative Cobb angle of 60 degrees. To reduce the postoperative CIB, the correction rate of the main and compensatory curves should be consistent. Meanwhile, lumbar motion segments should be preserved as much as possible to provide the ability to spontaneously compensate. Bao et al. [22] reported that postoperative CIB with pelvic fixation may not compensate spontaneously during follow-up, resulting in permanent fixation decompensation.

Preoperative CIB− patients had a postoperative CIB incidence of 60%. However, they had limited compensatory capacity, and none of them could compensate CB in the follow-up. However, from another perspective, CIB− patients achieved the greatest improvement in ΔCBD after PSF surgery. What is more, they had the lowest VAS back pain scores on average at the final follow-up. Even though the CBD in CIB− patients was more than 2 cm, the CSVL divided the curve of the thoracic and lumbar spine, and the center of gravity of the spine remained close to the CSVL. Thus, the trunk of the CIB− patients was mechanically stable, and the energy required to maintain the stability was minimal. This may be one of the reasons why the CIB− patients had less back pain. To improve the postoperative coronal balance, the main thoracolumbar curvature should be well corrected by osteotomy, and the thoracic compensatory curvature should not be corrected beyond the main curvature.

CIB+ patients had mechanical instability of the spine, which is similar to the Leaning Tower of Pisa. The postoperative CIB rate, which was 64.28%, had deteriorated at the final follow-up to 71.43%. The results show that CIB+ patients were the least able to compensate. Similar findings have been reported in previous studies of patients with congenital scoliosis and degenerative lumbar scoliosis. In a study of 118 patients with congenital thoracolumbar scoliosis, Liang et al. [23] reported that patients who lost compensation to the convex side preoperatively had a higher rate of coronal loss postoperatively. Bao et al. [24] reported that patients with degenerative lumbar scoliosis with type C coronal misalignment, CBD > 3 cm, and C7PL shifted toward the convex side were at greater risk of developing coronal imbalance after posterior osteotomy. Therefore, postoperative CIB needs to be emphasized when consulting with CIB+ patients. The correction of lumbar or lumbar-sacral compensatory curvature is the key to avoiding postoperative CIB in preoperative CIB+ patients. Contrary to CIB− patients, CIB+ patients should have a correction rate of the lumbar or lumbar-sacral compensatory curvature that is greater than that of the main thoracic curvature.

Lumbar or lumbar-sacral curvature is usually more rigid than the thoracic curvature in AdIS, resulting in greater difficulty in adequate correction. A surgical plan can be determined according to the flexibility of the spine and the training level of the surgeon.

The multiple-level asymmetrical Ponte osteotomy [15] is a safe and effective technique that reduces operation time, blood loss, and complications and offers an appropriate option to address the problems of rigid adult idiopathic scoliosis. Multiple-level asymmetrical Ponte osteotomy or interbody release makes a difference in improving the correction rate, especially for the rigid lumbar-sacral curve. Asymmetric pedicle subtraction osteotomy (APSO) [25,26], which has a higher correction capacity than posterior column osteotomy, is usually performed on the convex side of the main curve or lumbosacral fractional curvature. Asymmetrical transforaminal lumbar fusion (TLIF) [27,28] is suitable for the correction of lumbosacral fractional curvature. Implanting an intervertebral fusion cage on the concave side of lumbosacral fractional curvature can restore the lumbosacral tilt and achieve the function of correcting the balance. In addition, anterior approach release and intervertebral fusion, including lateral lumbar interbody fusion (LLIF) or oblique lumbar interbody fusion (OLIF) [29], can well restore the height of the intervertebral space, rearrange the sequence of vertebral bodies, and improve the coronal imbalance caused by lumbar scoliosis. During surgery, LIV should be leveled as much as possible to provide a homogeneous base for the whole spine. Postoperative back muscle training programs and bracing can also make a difference to CIB compensation [30,31,32].

The CIB− patients reported a lower back pain score than did the CIB+ patients. This can be explained by the CIB− patients’ trunk center of gravity being closer to the “cone of economy” [33,34] and the patients expending less energy to maintain trunk balance. Although only CIB− patients reported differences in pain score at the last follow-up, the classification method proposed in this study summarizes the rule of changes in postoperative coronal balance in the four types of patients, which provides a useful reference for predicting and preventing CIB after surgery in AdIS patients.

This is the first study to categorize the coronal balance of AdIS based on the apical vertebras distribution modifier and to propose a treatment algorithm aiming to avoid postoperative CIB. However, the limitations of this study should be acknowledged. Even though each type of included patient well represents the rule of coronal balance development, the sample size of this study is relatively small, and most of the patients were relatively young adults. Therefore, the patient inclusion bias cannot be ignored. In future studies, larger samples are needed for prospective studies to further validate the validity of this classification method and to improve the surgical algorithm. Whether this classification method can be extended to other types of deformity patients, including teenagers and the elderly, as well as congenital scoliosis and degenerative scoliosis patients, remains to be verified in future research.

## 5. Conclusions

In conclusion, the apical vertebras distribution modifier is an important supplement to the scoliosis coronal balance classification. It fully takes into account the patient’s center-of-gravity distribution, which can truly reflect the coronal loading balance from the perspective of biomechanics. The classification of coronal balance for AdIS based on the apical vertebras distribution modifier proposed in this study has good consistency and is simple and easy to use in the clinic. It can not only predict the long-term balance outcome of patients after surgery but also facilitate surgical plan-making according to the proposed surgical algorithm.

## Figures and Tables

**Table 1:** Demographical and radiographical parameters of the included patients (Mean values with standard deviations).

	Total	CB−	CB+	CIB−	CIB+	*p*-Value *
Number	80	10	46	10	14	-
Age (years)	25.97 ± 9.20 (18–45)	25.70 ± 6.96	24.96 ± 8.14	27.20 ± 7.88	29.50 ± 10.98	0.3648
Gender (M/F)	22/58	1/9	14/32	2/8	5/9	0.1755
Height (cm)	154.51 ± 8.35	160.75 ± 5.12	155.31 ± 8.92	150.10 ± 12.45	151.25 ± 6.34	0.2936
Weight (kg)	49.88 ± 7.68	54.38 ± 8.70	51.50 ± 9.50	49.10 ± 9.03	48.54 ± 8.01	0.8214
Preop Cobb Angle (°)	107.25 ± 21.11	99.02 ± 14.88	105.77 ± 20.84	115.03 ± 15.21	112.43 ± 25.88	0.2588
Preop CBD (mm)	20.05 ± 21.36	6.53 ± 4.95	9.38 ± 4.75	59.78 ± 18.08	36.38 ± 19.58	<0.0001
Follow-up (year)	3.76 ± 1.38 (2–8)	3.98 ± 1.48	3.62 ± 1.30	3.68 ± 1.14	4.11 ± 1.63	0.6401

* Comparison between four groups of patients.

**Table 2:** Comparison of radiological parameters of 80 AdIS patients according to the type of CB (Mean values with standard deviations).

	CB−	CB+	CIB−	CIB+	*p*-Value *
Number (%)	10	46	10	14	-
Preop Cobb Angle (°)	99.02 ± 14.88	105.77 ± 20.84	115.03 ± 15.21	112.43 ± 25.88	0.2588
Postop Cobb Angle (°)	54.09 ± 24.01	48.79 ± 24.05	65.52 ± 15.52	54.17 ± 25.56	0.2357
Fusion levels	12.20 ± 1.08	11.78 ± 1.39	13.30 ± 1.55	12.93 ± 1.71	0.0061
Preop CBD (mm)	6.53 ± 4.95	9.38 ± 4.75	59.78 ± 18.08	36.38 ± 19.58	<0.0001
△CBD (postop minus preop, − improvement, + aggravation) (mm)	+24.50 ± 19.77	+10.79 ± 14.01	−28.26 ± 19.39	−1.04 ± 15.97	<0.0001
△CBD (FU minus preop, − improvement, + aggravation) (mm)	+11.23 ± 9.64	+3.78 ± 12.07	−29.34 ± 14.59	−8.31 ± 13.62	<0.0001

* compared with preoperation.

**Table 3:** Coronal balance evolution of each group of patients.

Preop	CB−	CB+	CIB−	CIB+
Number (%)	10	46	10	14
Postop CIB	7 (70%)	23 (50%)	6 (60%)	9 (64.29%)
Final follow-up CIB	4 (40%)	13 (28.26%)	6 (60%)	10 (71.43%)

**Table 4:** VAS pain score, ODI, and SRS-22 patient questionnaire ^#^ evaluation of the included patients at the last follow-up (Mean values with standard deviations).

	CB−	CB+	CIB−	CIB+	p^1^	p^2^
VAS Back Pain	2.00 (1.29)	2.40 (2.08)	0.67 (0.94)	2.00 (1.30)	0.56	0.01 *
VAS Leg Pain	0.83 (0.69)	0.80 (1.63)	0.67 (0.94)	0.3 (0.47)	0.95	0.22
ODI%	7 (6)	10 (9)	5 (5)	13 (16)	0.32	0.14
Function/Activity	4.2 (0.38)	4.0 (0.54)	3.8 (0.34)	4.2 (0.63)	0.27	0.08
Pain	4.1 (0.52)	4.3 (0.30)	4.0 (0.36)	4.5 (0.43)	0.10	0.01 *
Self-image/Appearance	4.2 (0.43)	3.9 (0.85)	4.0 (0.56)	4.1 (0.38)	0.28	0.61
Mental health	4.0 (0.38)	4.1 (0.46)	4.4 (0.32)	4.5 (0.41)	0.52	0.53
Satisfaction with management	3.9 (0.87)	3.8 (0.94)	3.7 (0.45)	4.0 (0.32)	0.76	0.07

^#^ SRS-22 patient questionnaire is a commonly used patient-reported outcome measure of the quality of life that includes five dimensions: function/activity, pain, self-image/appearance, mental health, and satisfaction with management. Each dimension has a maximum score of 5, representing the best results. p^1^ represents group CB− vs. group CB+; p^2^ represents group CIB− vs. group CIB+. * Statistically significant.

**Table 5:** SF-36 * evaluation of the included patients at the last follow-up (Mean values with standard deviations).

	SF-36									
	PF	RP	BP	GH	VT	SF	RE	MH	HT	Total
CB−	87.50 (4.79)	100 (0.00)	83.33 (8.69)	70.83 (13.48)	69.17 (7.31)	118.75 (9.55)	88.89 (15.71)	73.33 (12.15)	75.00 (25.00)	766.81 (71.17)
CB+	84.25 (16.07)	60.00 (39.84)	85.80 (14.18)	67.05 (25.75)	63.50 (15.34)	109.38 (19.31)	71.67 (38.41)	69.20 (18.85)	75.00 (23.72)	685.84 (158.39)
CIB−	83.33 (4.71)	58.33 (31.18)	79.33 (4.11)	68.00 (17.38)	56.67 (14.34)	95.83 (15.59)	55.56 (41.57)	58.67 (18.57)	66.67 (23.57)	622.39 (88.15)
CIB+	88.33 (7.45)	87.50 (27.95)	80.67 (16.68)	68.00 (17.99)	60.00 (25.66)	89.58 (26.43)	61.11 (40.45)	62.67 (22.94)	66.67 (27.64)	664.53 (146.81)
*p*-value	0.88	0.07	0.78	0.99	0.69	0.50	0.50	0.62	0.86	0.46

* The SF-36 is a 36-item self-report measure of health-related quality of life. It has eight subscales measuring different domains of health-related quality of life: physical functioning (PF), role-physical (RP), bodily pain (BP), general health (GH), vitality (VT), social functioning (SF), role-emotional (RE), and mental health (MH). Each item has a maximum score of 100. The higher the score, the better the result.

## Data Availability

The data presented in this study are available on request from the corresponding author.

## Supplementary Materials

The following supporting information can be downloaded at: https://www.mdpi.com/article/10.3390/jpm13060897/s1, Table S1: Distribution of LIV and UIV; Table S2: Reliability test of consistency between different observers (n = 80); Table S3: Repeatability analysis for the same observer (n = 80).
