# Supplementary material for: Apical Vertebras Distribution Modifier for Coronal Balance Classification in Adult Idiopathic Scoliosis
Source: J Pers Med. 2023 May 26;13(6):897. doi: 10.3390/jpm13060897 (PMC10301802; doi:10.3390/jpm13060897)
Supplement: Supplementary file 1 [file jpm-13-00897-s001.zip › jpm-2279050-supplementary.pdf]

Supplemental Table S1. Distribution of LIV and UIV.

UIV	Number of cases	LIV	Number of cases
T1	1	T12	1
T2	24	L1	1
T3	27	L2	10
T4	15	L3	28
T5	6	L4	27
T6	3	L5	13
T7	2		
T8	1		
T9	1		

Supplemental Table S2. Reliability test of consistency between different observers (n=80).

	CB-	CB+	CIB-	CIB+	Kappa value
Observer 1	10	46	10	14	0.959
Observer 2	11	45	9	15	

Supplemental Table S3. Repeatability analysis for the same observer (n=80).

	CB-	CB+	CIB-	CIB+	Kappa value
First classification	10	46	10	14	0.979
Second classification	10	46	11	13	
